# Supplementary material for: Co-occurrence networks reveal more complexity than community composition in resistance and resilience of microbial communities
Source: Nat Commun. 2022 Jul 5;13:3867. doi: 10.1038/s41467-022-31343-y (PMC9256619; doi:10.1038/s41467-022-31343-y)
Supplement: Supplementary file 1 — Supplementary Information [file 41467_2022_31343_MOESM1_ESM.pdf]

Supplementary Information for "Co-occurrence Networks Reveal More Complexity Than Community Composition in Resistance and Resilience of Microbial Communities" by Gao et al.

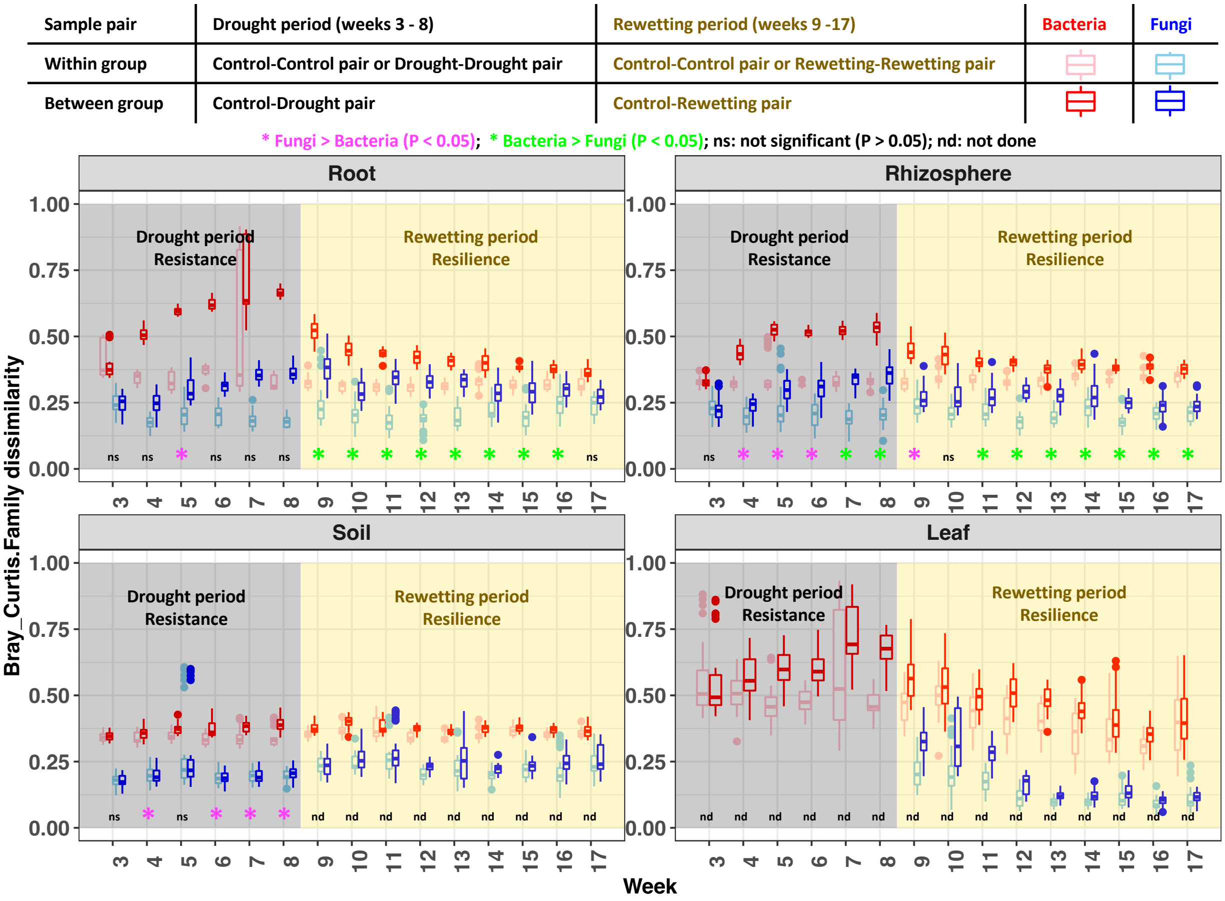

Supplementary Fig. 1. Resistance and resilience of bacterial OTUs composition and fungal family composition. Note the results at fungal OTU levels can be found in Fig. 1. Bray-Curtis dissimilarities were computed for bacterial and fungal communities of four compartments (root, rhizosphere, soil, leaf) of n=12 biologically independent plots examined over 17 weeks. The boxes represent the 25th–75th percentiles (with the median as a horizontal line) and the whiskers show the 10th–90th percentiles. Ecological resistance to drought stress is detected by comparing compositional dissimilarity of between-group pairs (control-drought pairs) against within-group pairs (control-control pairs and drought-drought pairs) at each of the droughted weeks (weeks 3 - 8, the grey shaded area) (*p < 0.05, adjusted by Bonferroni method; unpaired t-test, two-sided). Ecological resilience to rewetting is detected by assessing, from before to after rewetting, the change in the difference of compositional dissimilarity between within-group pairs and between-

group pairs. Here, the point just before rewetting was week 8 and the points after rewetting were weeks 9 – 17 (the gold shaded area). In most cases, the results of fungal families and OTUs are largely consistent. Different family and OTUs results were detected in four points where significances detected by OTUs were not detected by family (root, week 4 and 17), or significances detected by family were not detected by OTUs (rhizosphere, weeks 7 and 8). We report only results that are robust across these two conditions.

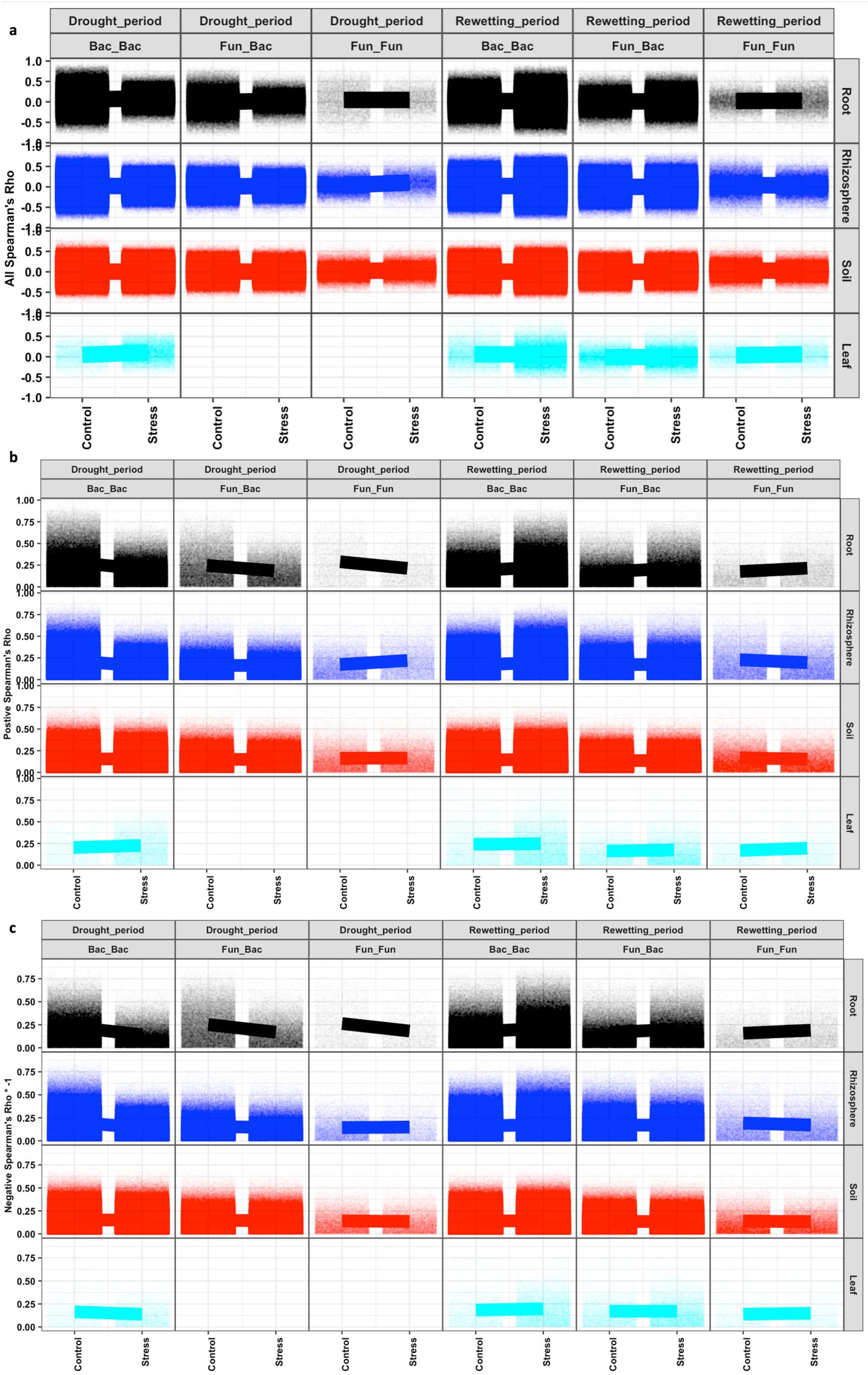

Supplementary Fig. 2 Pairwise Bacterial-Bacterial (Bac-Bac), Fungal-Bacterial (Fun_Bac), and Fungal-Fungal (Fun_Fun) correlations in drought period and rewetting period in root, rhizosphere, soil and leaf. This figure illustrates the data points underlying the lines of each compartment and each correlation type shown in Fig. 2.

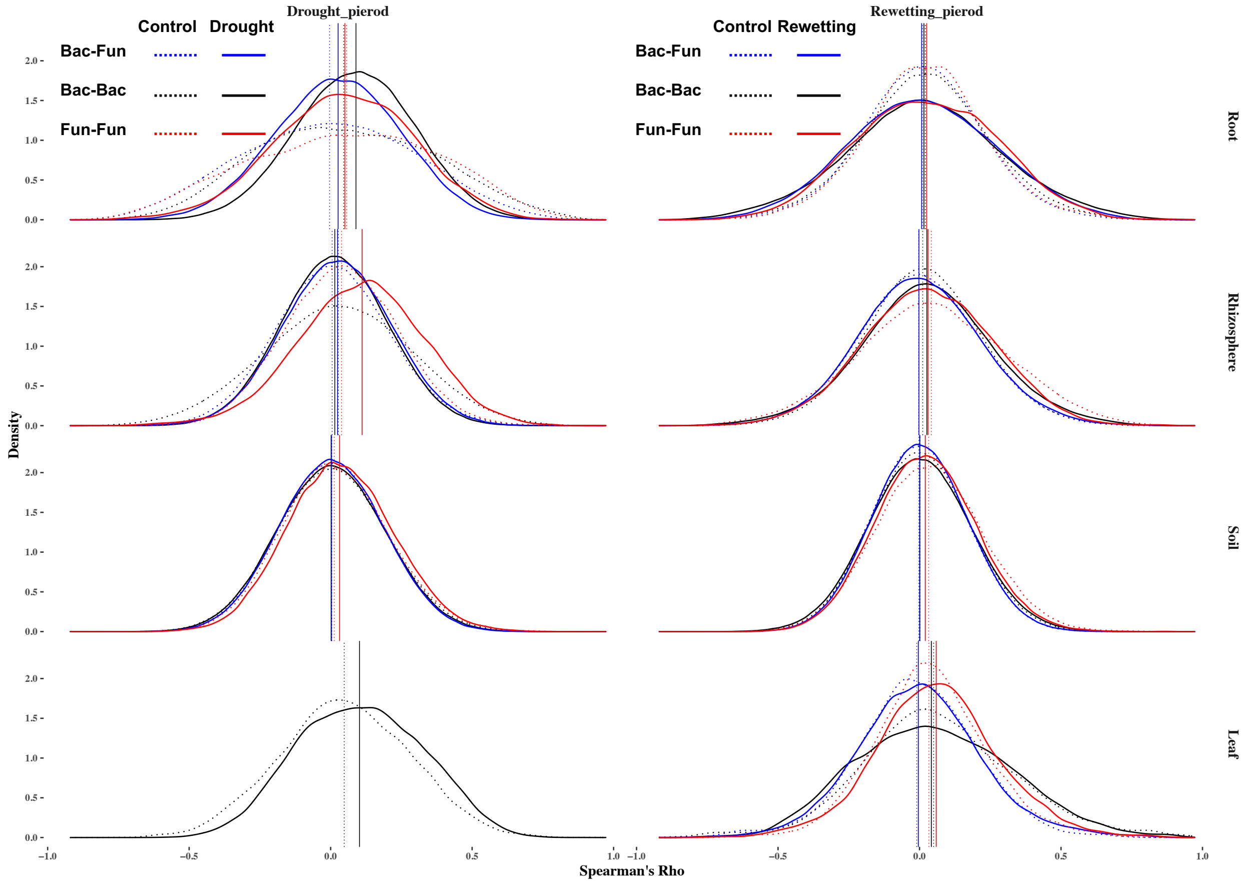

Supplementary Fig. 3 Frequency distributions of all correlations between microbial taxa as assessed by Spearman's Rho. In general, drought decreased and rewetting increased the strength of correlations. However, obvious exceptions are seen for the effect of drought on fungus-fungus correlations in rhizosphere and bacterium-bacterium correlations in leaf.

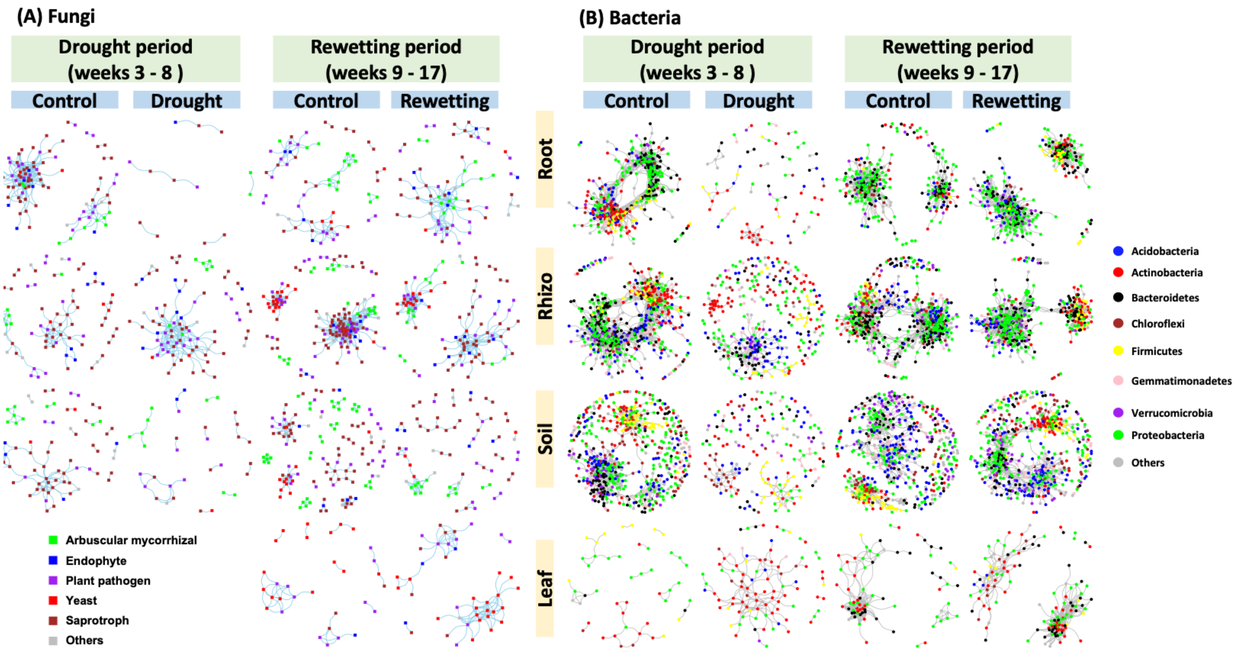

Supplementary Fig. 4 Subnetworks of significant positive Spearman correlations (A) between fungal taxa and (B) between bacterial taxa. (A) Subnetworks of significant positive correlations between fungal OTUs. The FF co-occurrence in the rhizosphere is enhanced by drought, although it is disrupted in root. Re-watering caused recovery of the FF network, with overcompensation in root and a lag in rhizosphere and soil. (B) Subnetworks of significant correlations between bacterial OTUs. The BB co-occurrence in leaf is enhanced by drought, although it is disrupted in root, rhizosphere and soil. Re-watering caused recovery of the BB network.

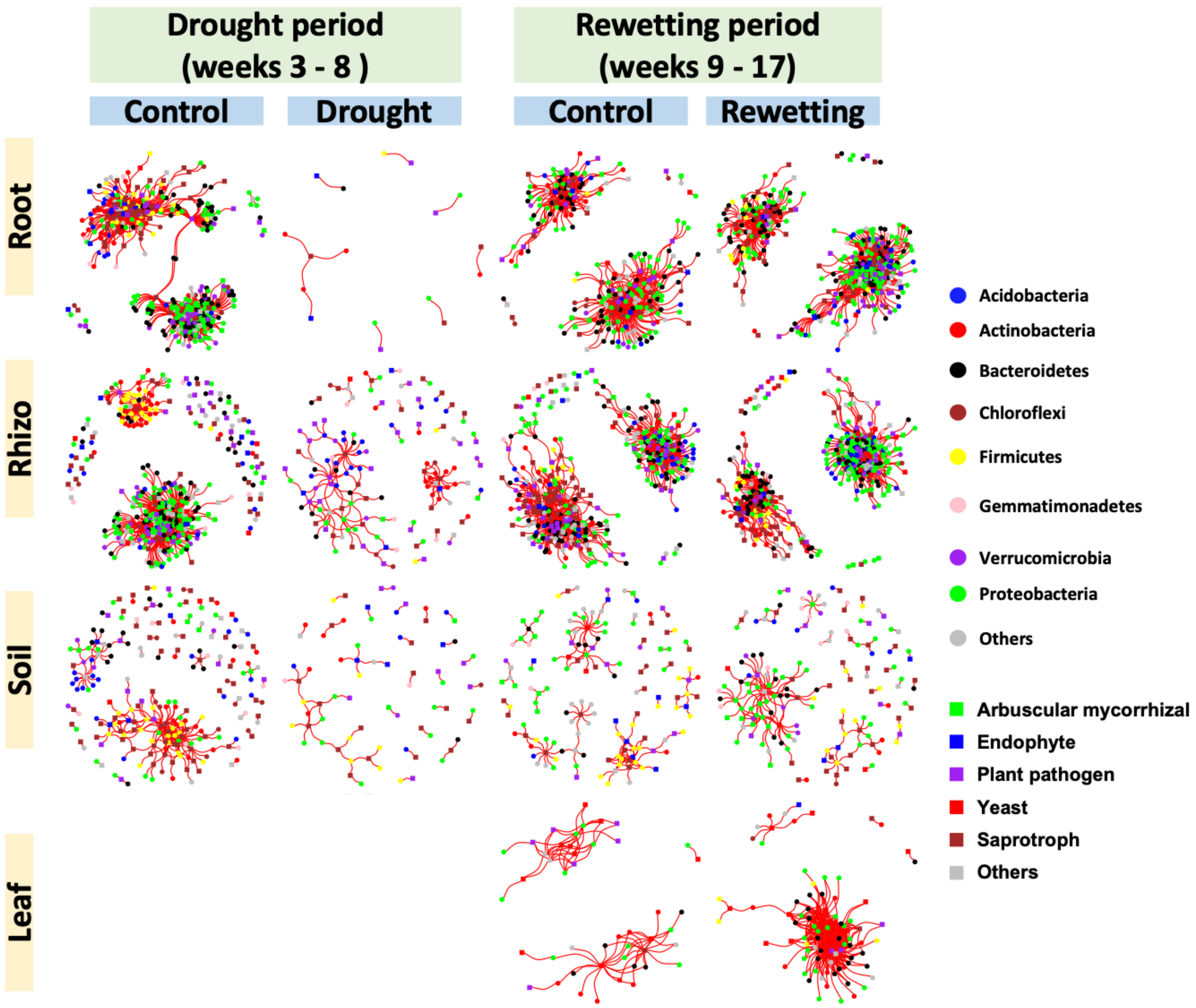

Supplementary Fig. 5 Subnetworks of significant correlations between bacterial and fungal OTUs. The BF co-occurrence in root, rhizosphere and soil are drastically disrupted by pre-flowering drought. Re-watering caused recovery of BB network, with overcompensation in root and leaf.

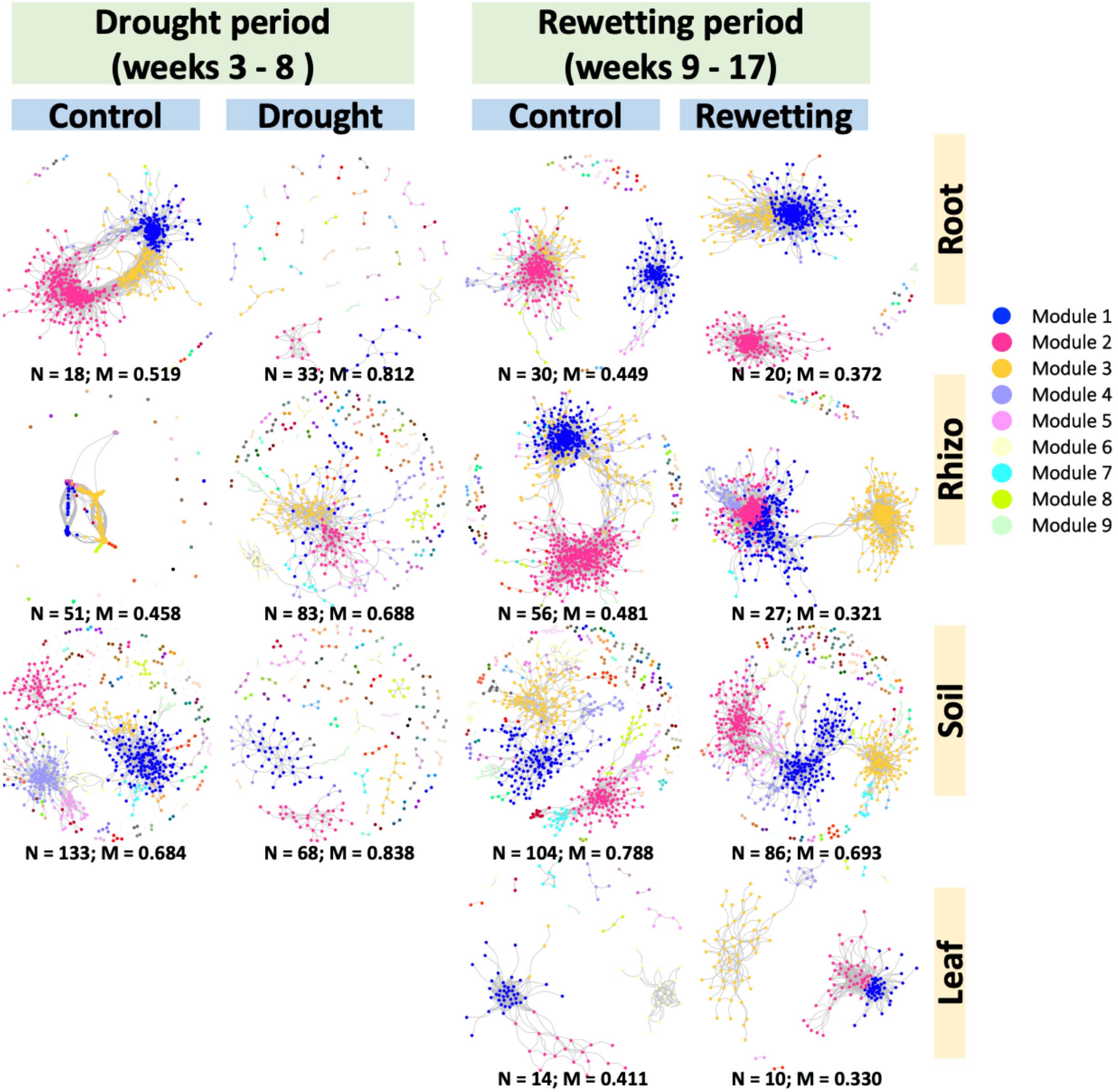

Supplementary Fig. 6 Modularity of networks of significant positive cross-taxonomic group correlations (bacteria and fungi). Network modules are detected by the cluster_fast_greedy method and demonstrated by different colors. N: the number of modules detected. M: modularity. In general, drought increased, and rewatering decreased the modularity of cross-domain co-occurrence networks; except for the modularity BB network in leaf that was decrease by pre-flowering drought.

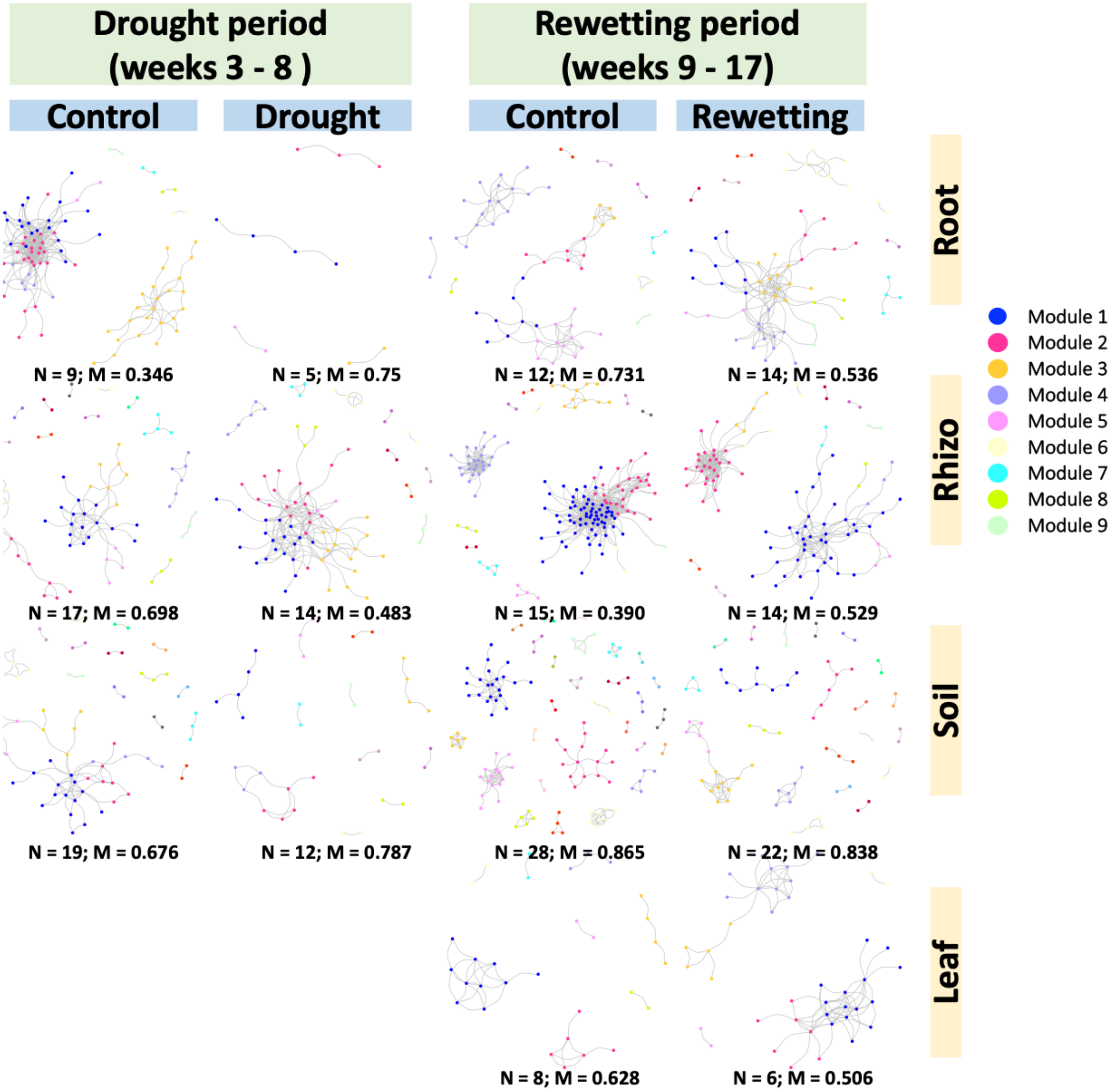

Supplementary Fig. 7 Modularity of networks of significant positive fungal-fungal correlations. Network modules are detected by the cluster_fast_greedy method and demonstrated by different colors. N: the number of modules detected. M: modularity.

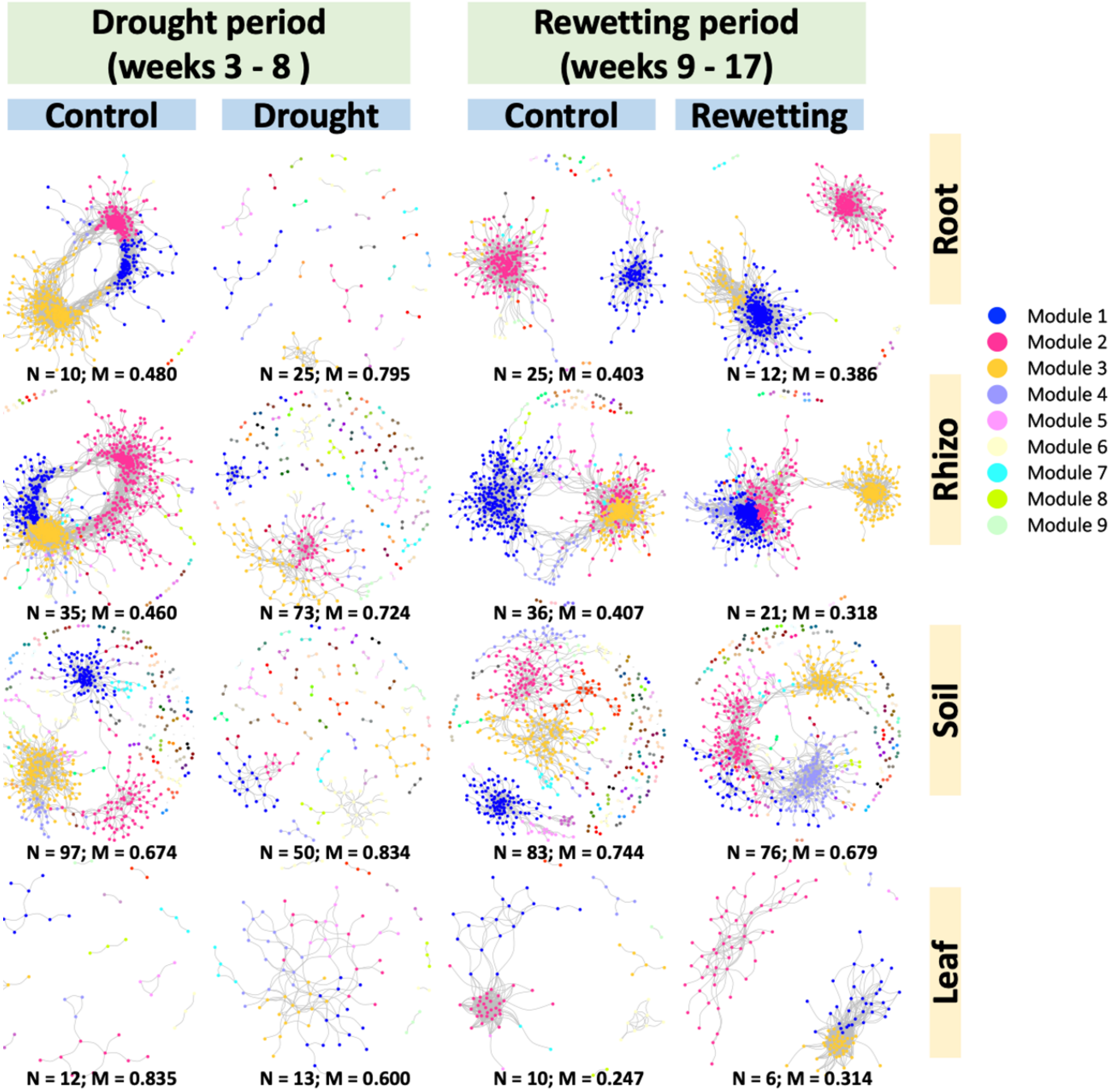

Supplementary Fig. 8 Modularity of networks of significant positive bacterial-bacterial correlations. Network modules are detected by the cluster_fast_greedy method and demonstrated by different colors. N: the number of modules detected. M: modularity.

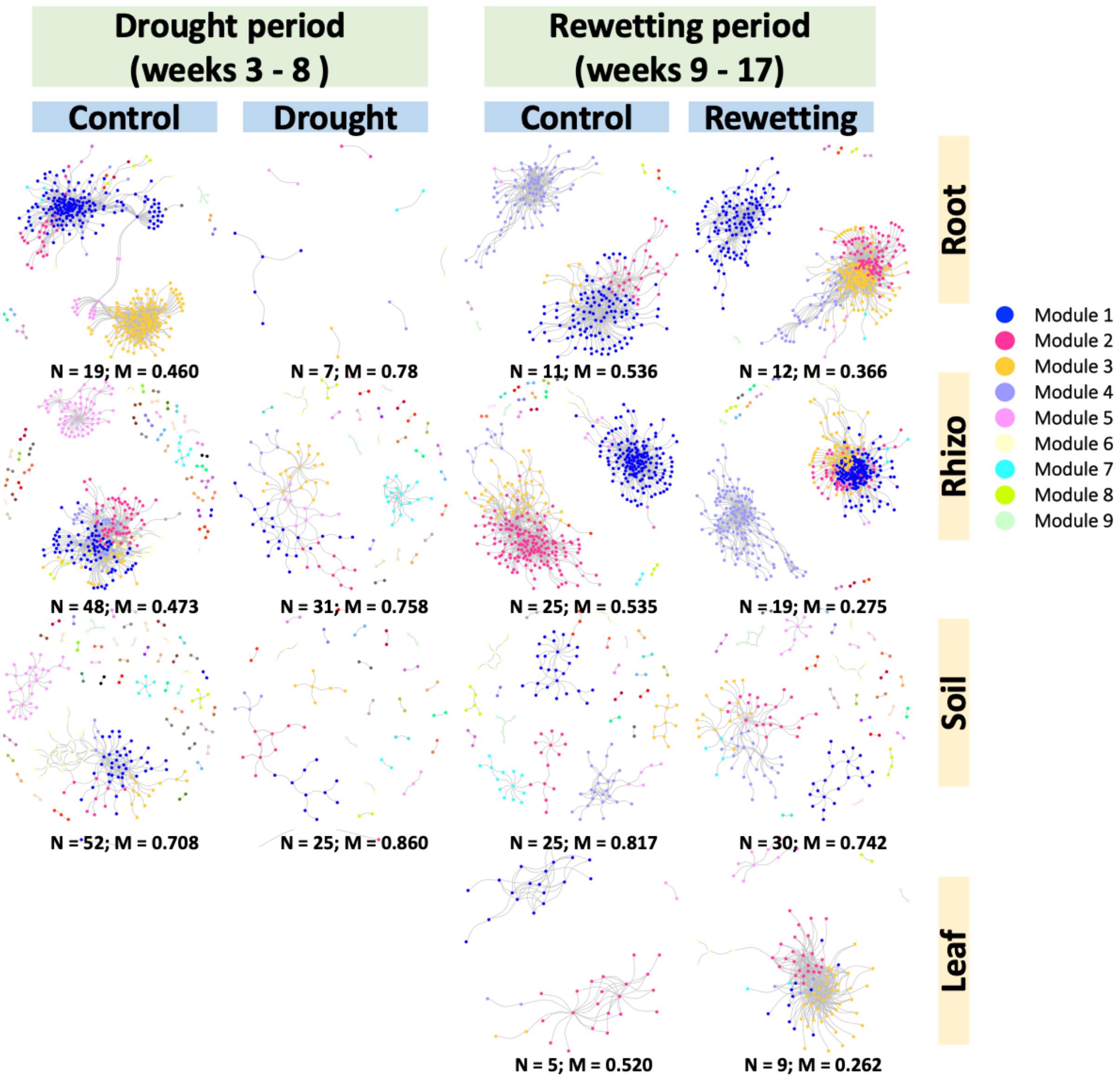

Supplementary Fig. 9 Modularity of networks of significant positive fungal-bacterial correlations. Network modules are detected by the cluster_fast_greedy method and demonstrated by different colors. N: the number of modules detected. M: modularity.

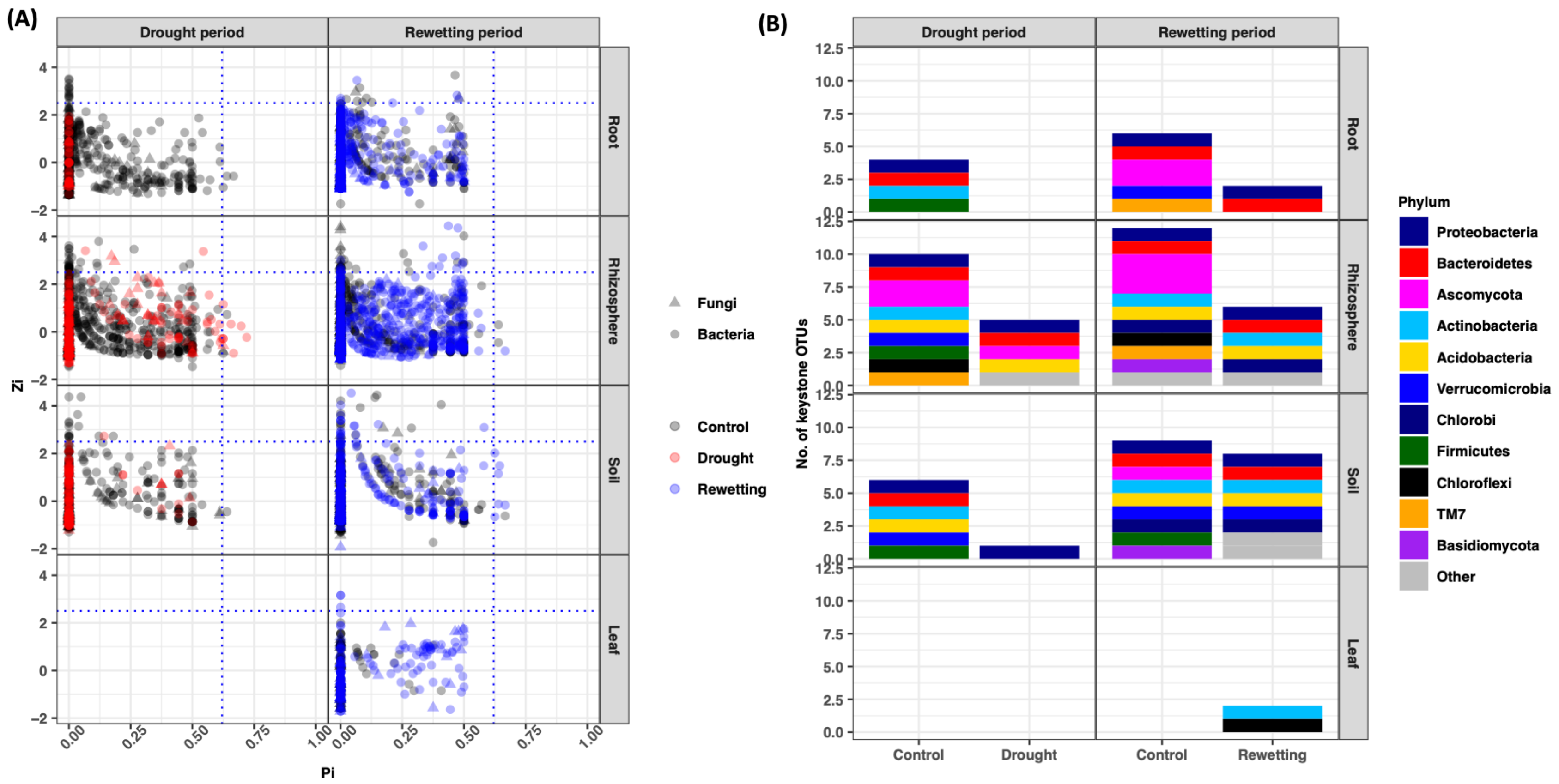

Supplementary Fig. 10 Detection by connectivity of putative keystone taxa and their phyla. (A) Recognition of putative keystone taxa by connectivity within-modules (Zi) and among-modules (Pi). Module hubs have Zi > 2.5, connectors have Pi > 0.62, and network hubs have Zi > 2.5 and Pi > 0.62. (B) Phyla and abundance of putative keystone taxa.

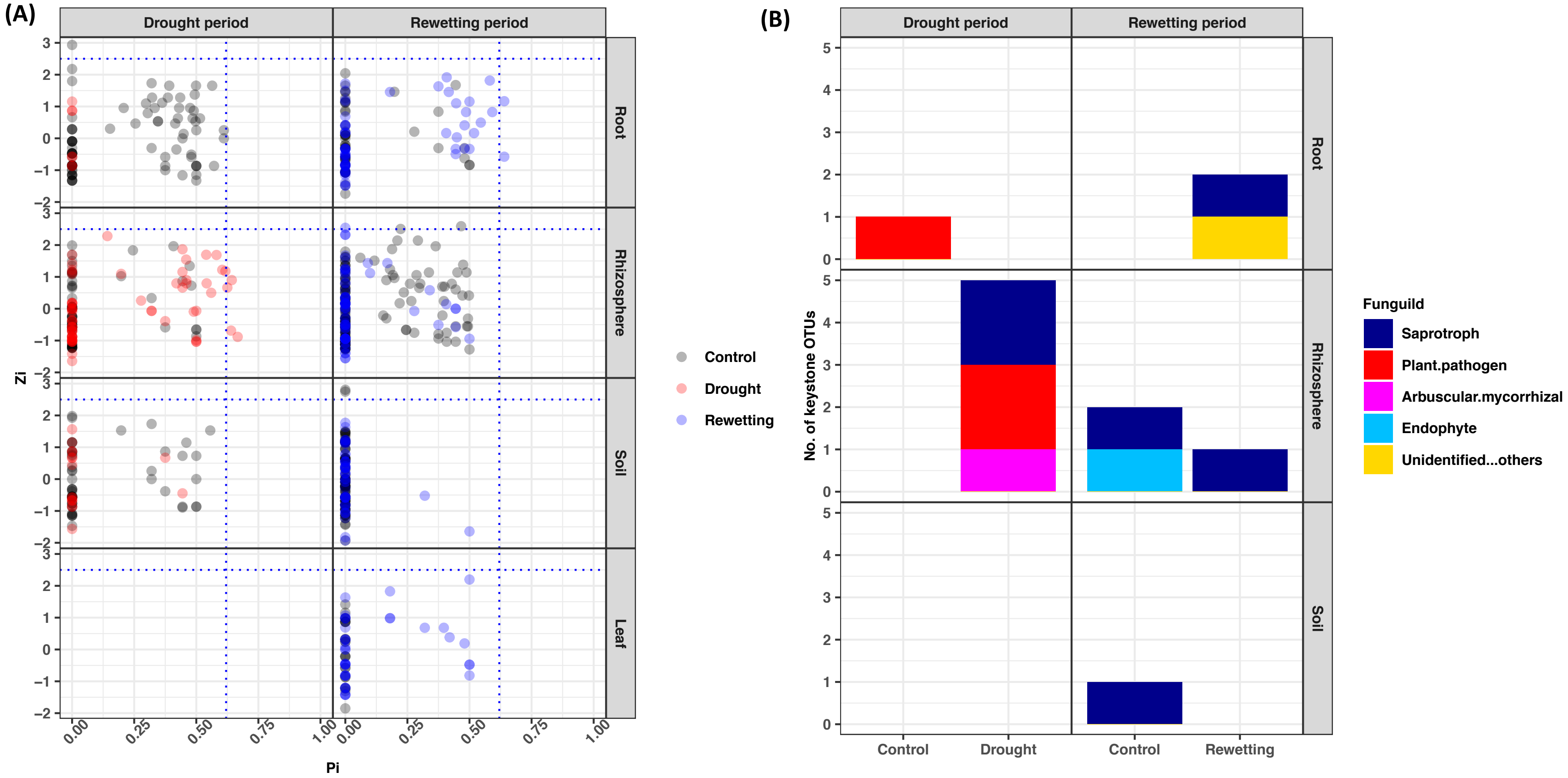

Supplementary Fig. 11 Detection by connectivity of putative keystone taxa and their functional guilds of fungal-fungal network. (A) Recognition of putative keystone taxa by connectivity within-modules (Zi) and among-modules (Pi). Module hubs have Zi > 2.5, connectors have Pi > 0.62, and network hubs have Zi > 2.5 and Pi > 0.62. (B) Fungal functional guilds and number of putative keystone taxa.

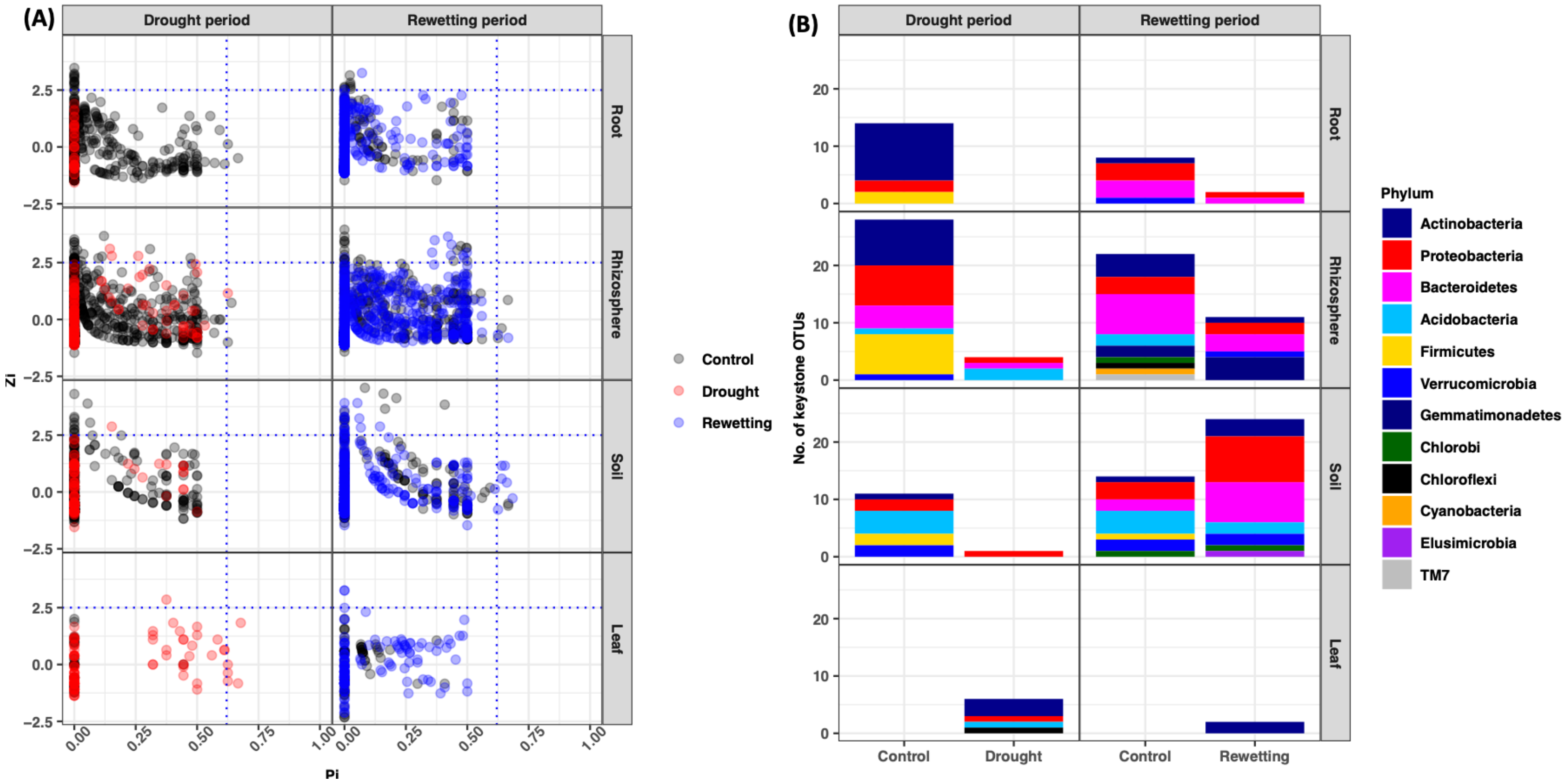

Supplementary Fig. 12 Detection by connectivity of putative keystone taxa and their phylum of bacterial-bacterial network. (A) Recognition of putative keystone taxa by connectivity within-modules (Zi) and among-modules (Pi). Module hubs have Zi > 2.5, connectors have Pi > 0.62, and network hubs have Zi > 2.5 and Pi > 0.62. (B) Bacterial phylum and number of putative keystone taxa.

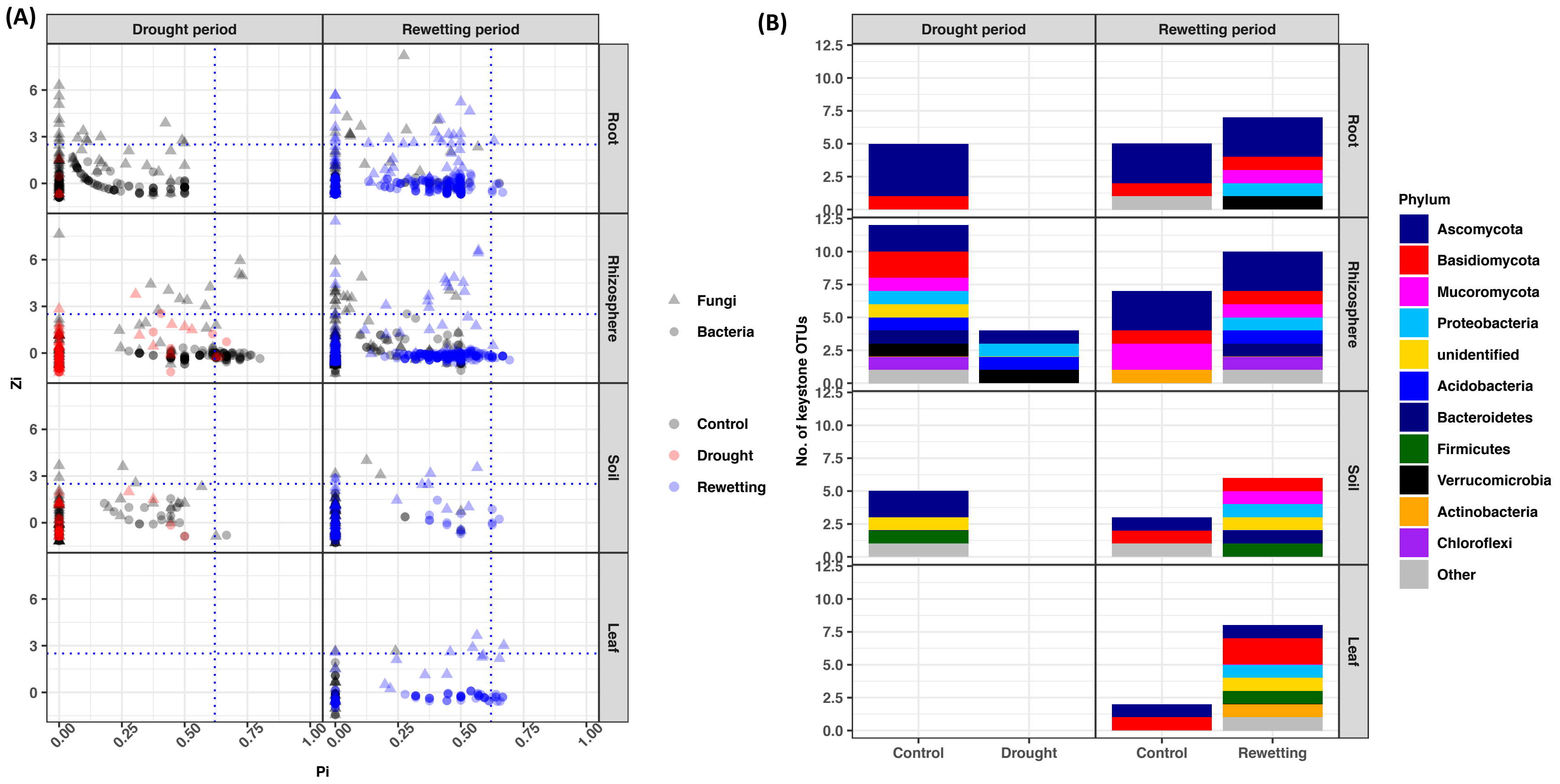

Supplementary Fig. 13 Detection by connectivity of putative keystone taxa and their phylum of fungal-bacterial network. (A) Recognition of putative keystone taxa by connectivity within-modules (Zi) and among-modules (Pi). Modules hubs have Zi > 2.5, connectors have Pi > 0.62, and network hubs have Zi > 2.5 and Pi > 0.62. (B) Fungal and bacterial phylum and number of putative keystone taxa.

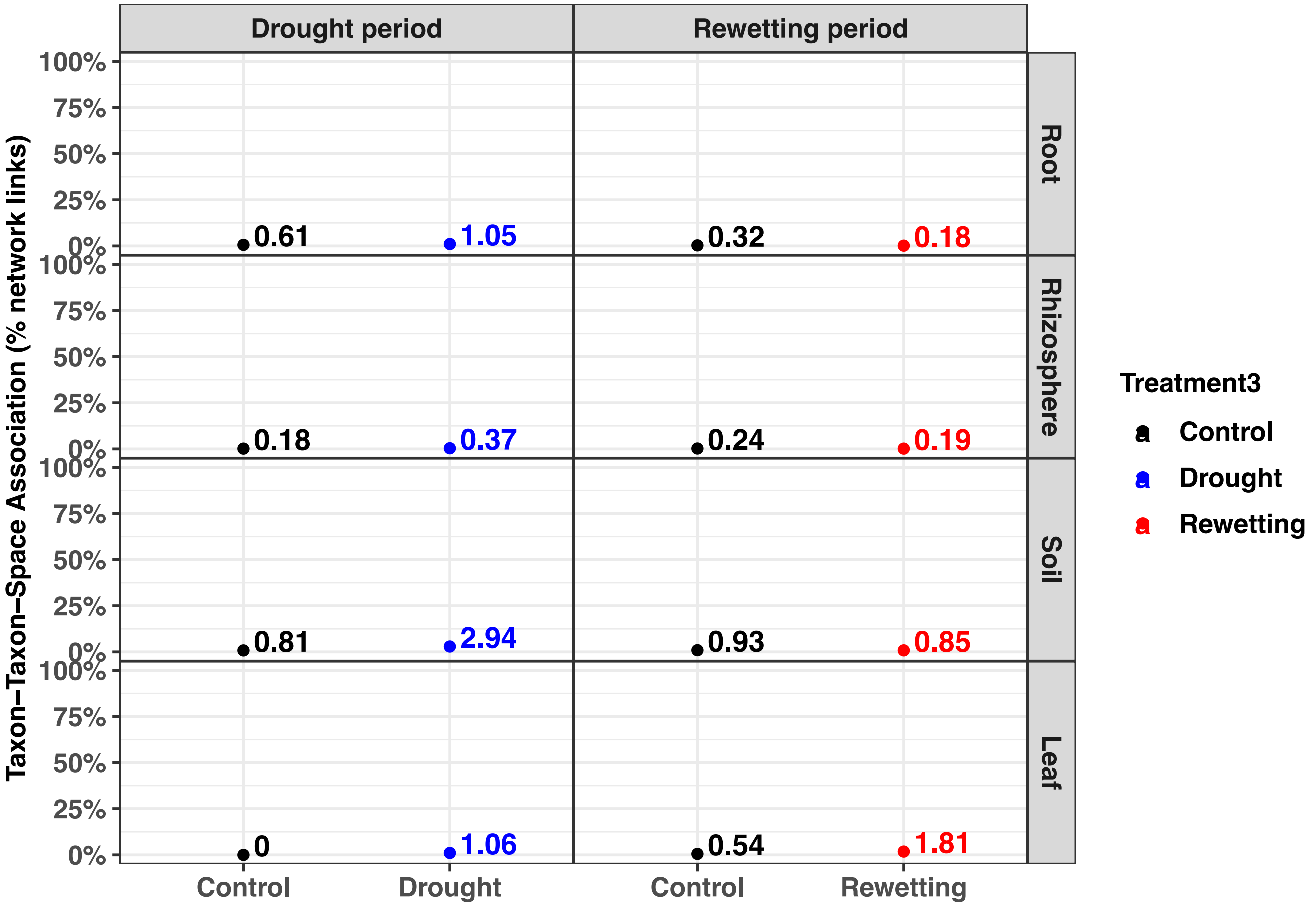

Supplementary Fig. 14 Proportion of taxon-taxon associations related to dispersal limitation. For each of taxon-taxon pair in the co-occurrence network, dispersal limitation was regarded as the driver if both taxa showed significant correlation with spatial distance.

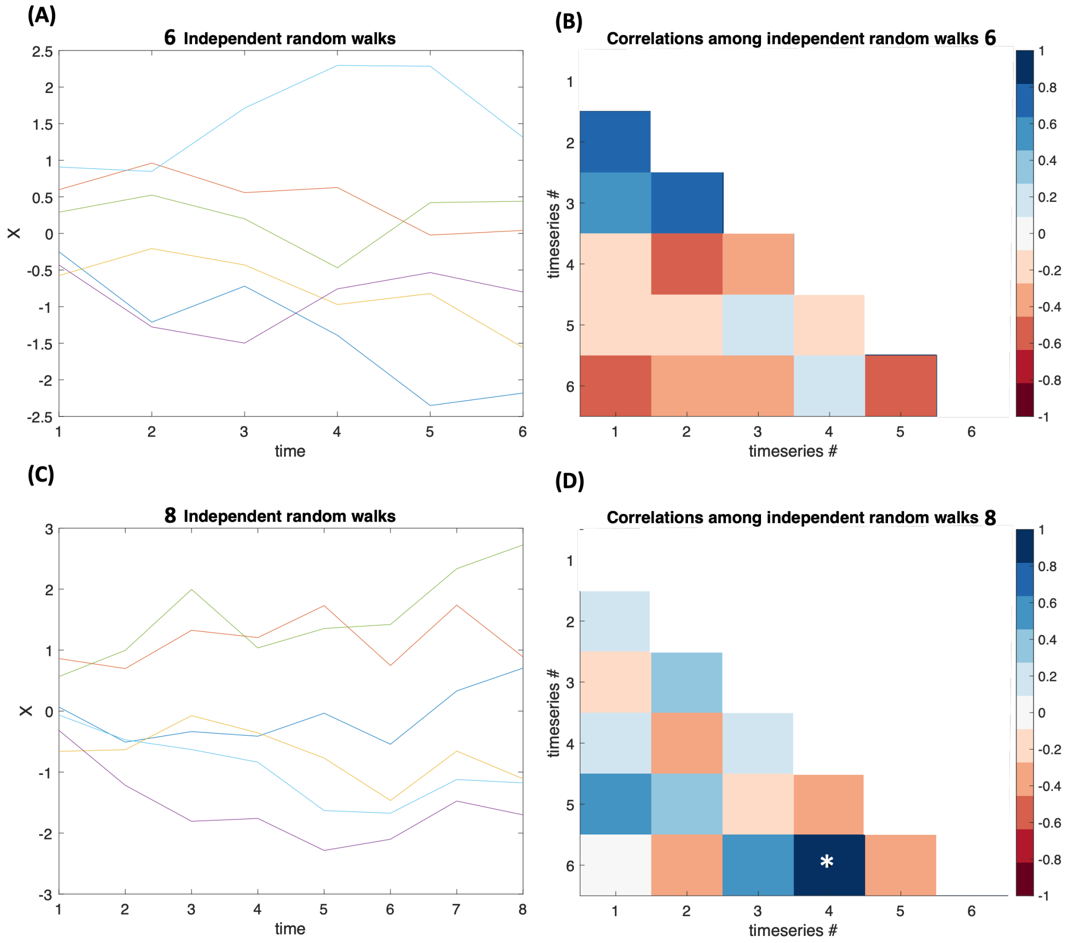

Supplementary Fig. 15 Minimal spurious association was detected in using the approach of Coenen et al 2020 [1] from 6 and 8 independent random walks over 6 temporal series. The analysis was repeated 10 times and results from one run are shown here. (A) Six time-series of six independent random walks mimicking the drought period. (B) For the 15 correlations among six time series of six independent random walks, at most 0-1 significant spurious associations were detected (none were found in this example). (C) Six time-series of eight independent random walks mimicking the rewetting period. (D) For the 15 correlations among six time series of eight independent random walks, at most 1-3 significant spurious associations were detected (The one in this run is marked with an asterisk in the example).

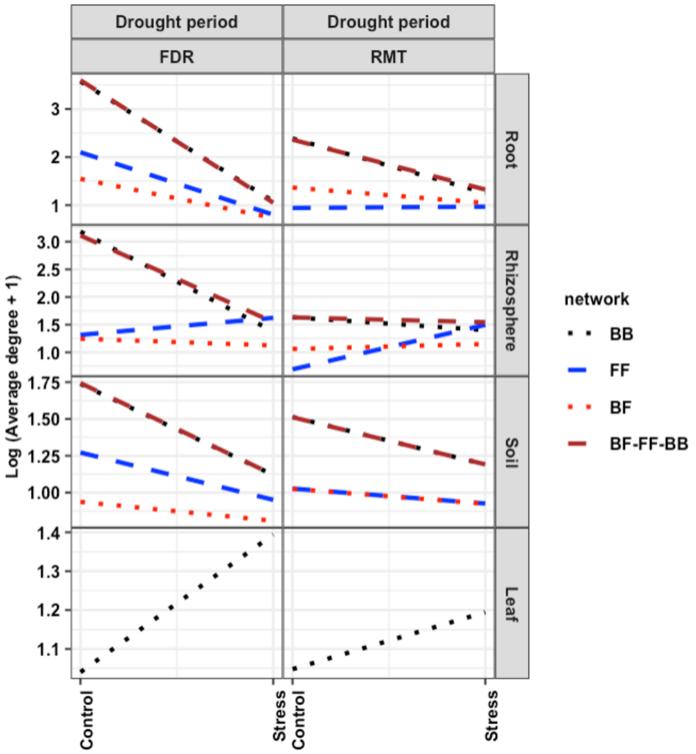

Supplementary Fig. 16 Consistent responses to drought of average degree of association networks based on Spearman correlations as filtered by either the false discovery rate (FDR) or random matrix theory (RMT) approach. Note that in only one case, roots, is there disagreement where the FF network showed disruption using the FDR approach but was unchanged using the RMT approach.

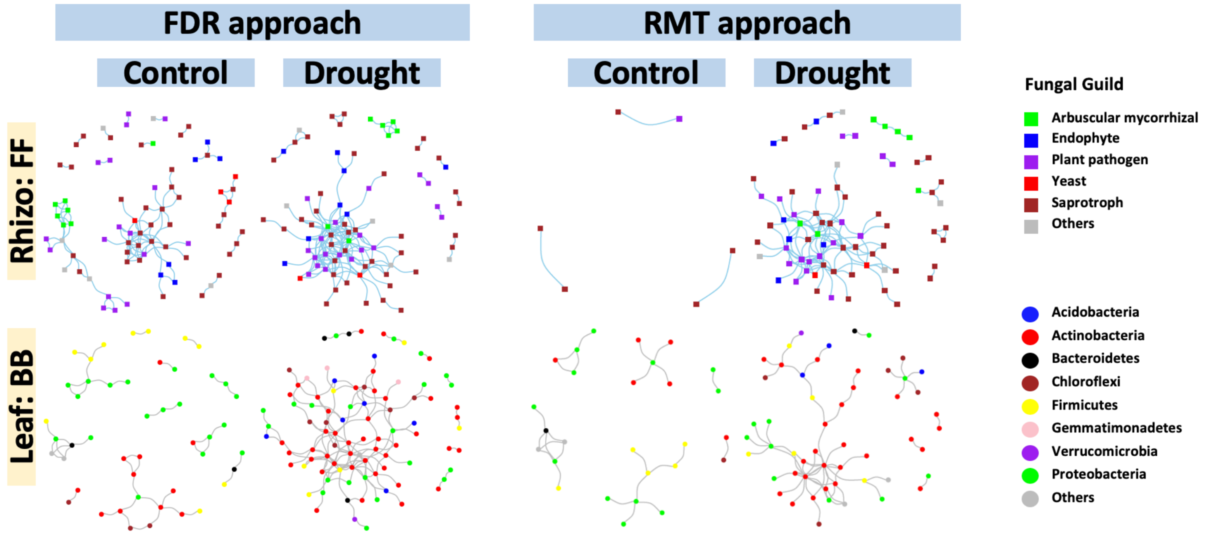

Supplementary Fig. 17 Spearman Rho co-occurrence networks of rhizosphere fungi and leaf bacteria were dramatically strengthened by drought, whether measured by FDR- or RMT-based approach.

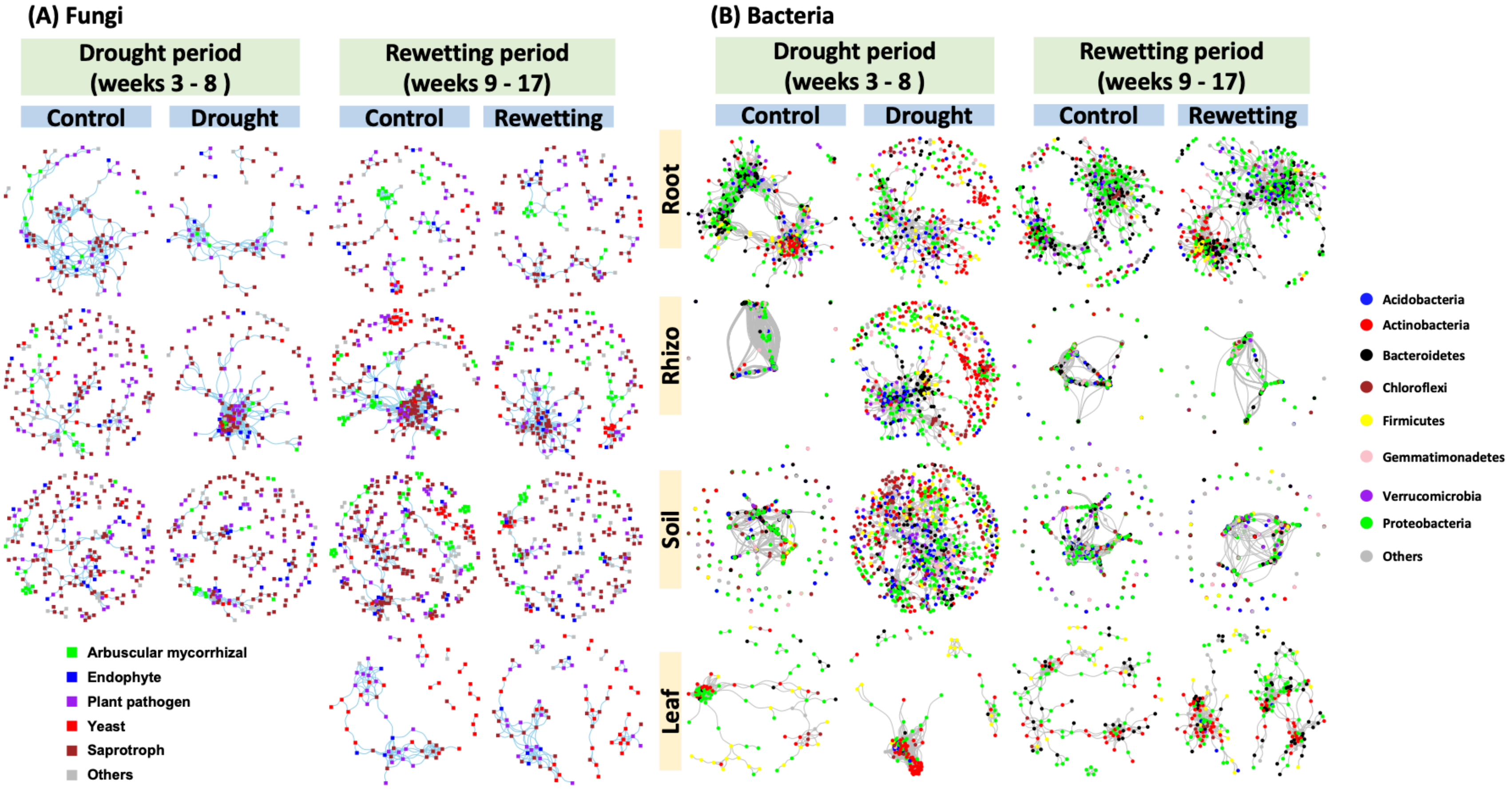

Supplementary Fig. 18 Co-occurrence network using the Pearson method. (A) The fungal co-occurrence network in the rhizosphere is enhanced by drought, although it is disrupted in root. (B) The bacterial co-occurrence network in leaf is enhanced by drought, although it is disrupted in root, rhizosphere and soil. Rewetting caused recovery of both fungal and bacterial networks.

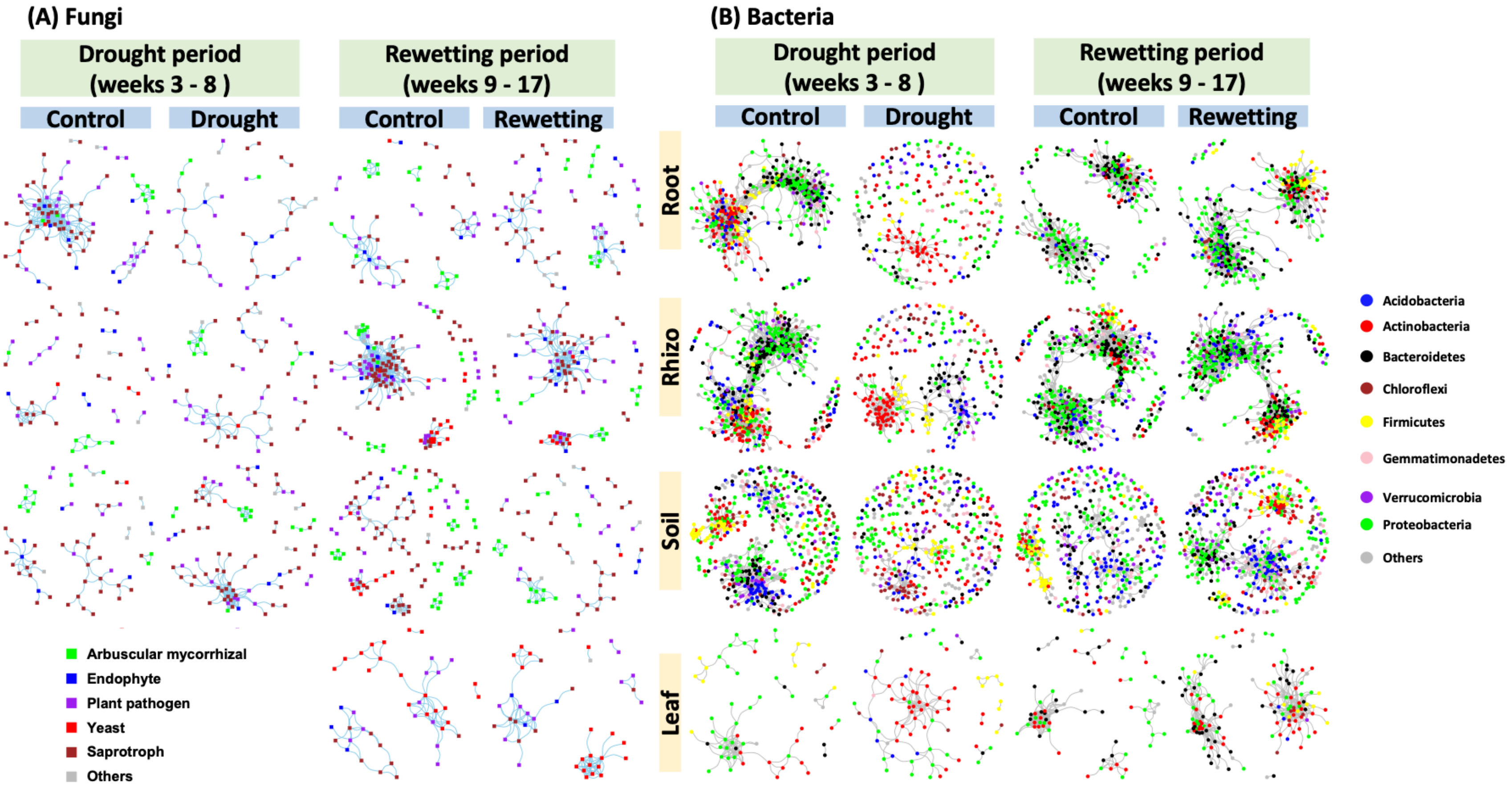

Supplementary Fig. 19 Co-occurrence network using the CoDa method. (A) The fungal co-occurrence network in the rhizosphere is enhanced by drought, although it is disrupted in root. (B) The bacterial co-occurrence network in leaf is enhanced by drought, although it is disrupted in root, rhizosphere and soil. Rewetting caused recovery of both fungal and bacterial networks.

Supplementary Table 1 Resistance & resilience of microbial community in response to drought disturbance

Study	Drought treatment	Resistance (drought effect)	Re-wetting treatment	Resilience (re-wetting)	Note	Habitat
2	After one season of normal growth, during the second season, rain exclusion by placing transparent rain covers over the pots (three weeks)	Bacterial community was more strongly affected by drought than fungal community. Both significant	One week or two months after drought	Control v. stress similarity remain significantly lower at re-watering than before drought for bacterial but not fungal community	16S ITS	Soil / Grassland mesocosms
3	Natural dry-down in from wet April to dry Sep in California (lack of always watered control)	Bacterial but not fungal community as strongly affected by drought	Watering and sampling 2h later	Bacterial community return to the status of wet April<br>Fungal community not changed directionally		Soil / Grasslands
4	Grassland (April) soil subjected to three treatments: weekly watering (four month), weekly watering (2month) followed by no water (2 month), no watering (4 month)	Bacterial communities not different among three treatments<br>Fungal community (RNA but not DNA) different between wet and dry treatment	Watering and sampling 2h later	Re-wetting affected bacterial community (RNA but not DNA)<br>Re-wetting do not affect fungal community		Soil / greenhouse
5	Rainout shelter (-66% precipitation)	Drought significantly altered the community composition of soil bacteria<br>and, to a lesser extent, fungi (ns) in grasslands from two continents.				
6	Monsoon Precipitation	Fungal community is more affected by Monsoon Precipitation than bacterial community (note the confounding effect of fungal			454 sequencing of 16S and ITS	

		succession driven by vegetation dynamics)				
7 8 9		Greater ratio of fungi to bacteria correlated with decreased resistance		A greater ratio of fungi to bacteria correlated increased resilience.	PLFA	

Supplementary Table 2. Results of t tests on the resistance and resilience between bacterial community and fungal community at levels of operational taxonomic unit (OTU) and family. The P values were adjusted by Bonferroni method

Compartment	Week	Property	Level of fungi	t	Padj
Root	3	Resistance	Family	-0.793	1.0E+00
Root	3	Resistance	OTU	-1.281	1.0E+00
Root	4	Resistance	Family	-1.135	1.0E+00
Root	4	Resistance	OTU	-4.704	1.5E-04
Root	5	Resistance	Family	-6.099	1.7E-05
Root	5	Resistance	OTU	-7.977	1.8E-07
Root	6	Resistance	OTU	0.785	1.0E+00
Root	6	Resistance	Family	-2.483	2.1E-01
Root	7	Resistance	Family	0.186	1.0E+00
Root	7	Resistance	OTU	1.562	7.6E-01
Root	8	Resistance	OTU	-0.023	1.0E+00
Root	8	Resistance	Family	-1.908	4.7E-01
Root	9	Resilience	Family	15.087	2.9E-41
Root	9	Resilience	OTU	17.084	3.9E-52
Root	10	Resilience	Family	6.257	7.8E-09
Root	10	Resilience	OTU	11.722	2.2E-27
Root	11	Resilience	OTU	27.278	3.5E-95
Root	11	Resilience	Family	47.572	5.2E-160
Root	12	Resilience	OTU	28.094	6.1E-115
Root	12	Resilience	Family	45.034	8.7E-196
Root	13	Resilience	OTU	23.005	8.8E-73
Root	13	Resilience	Family	50.493	3.7E-173
Root	14	Resilience	OTU	6.006	3.1E-08
Root	14	Resilience	Family	16.519	5.0E-47
Root	15	Resilience	Family	24.543	2.7E-79
Root	15	Resilience	OTU	25.289	5.9E-85
Root	16	Resilience	Family	10.123	8.2E-21
Root	16	Resilience	OTU	20.748	3.1E-71
Root	17	Resilience	Family	2.513	1.1E-01
Root	17	Resilience	OTU	3.019	2.4E-02
Rhizosphere	3	Resistance	Family	-1.588	7.2E-01
Rhizosphere	3	Resistance	OTU	-2.644	6.5E-02
Rhizosphere	4	Resistance	Family	-4.735	1.1E-04
Rhizosphere	4	Resistance	OTU	-7.491	1.0E-09
Rhizosphere	5	Resistance	Family	-4.719	1.7E-04

Rhizosphere	5	Resistance	OTU	-12.059	4.9E-15
Rhizosphere	6	Resistance	Family	-4.130	1.6E-03
Rhizosphere	6	Resistance	OTU	-8.976	1.8E-09
Rhizosphere	7	Resistance	OTU	-2.597	7.4E-02
Rhizosphere	7	Resistance	Family	6.384	4.7E-07
Rhizosphere	8	Resistance	OTU	-1.179	1.0E+00
Rhizosphere	8	Resistance	Family	2.926	3.1E-02
Rhizosphere	9	Resilience	OTU	-30.498	5.9E-167
Rhizosphere	9	Resilience	Family	-35.850	1.5E-214
Rhizosphere	10	Resilience	OTU	-1.940	4.7E-01
Rhizosphere	10	Resilience	Family	-2.514	1.1E-01
Rhizosphere	11	Resilience	Family	9.162	2.0E-18
Rhizosphere	11	Resilience	OTU	13.518	4.2E-38
Rhizosphere	12	Resilience	OTU	24.078	1.9E-102
Rhizosphere	12	Resilience	Family	38.143	1.1E-202
Rhizosphere	13	Resilience	Family	47.101	0.0E+00
Rhizosphere	13	Resilience	OTU	49.197	0.0E+00
Rhizosphere	14	Resilience	OTU	5.625	1.9E-07
Rhizosphere	14	Resilience	Family	8.502	3.9E-16
Rhizosphere	15	Resilience	OTU	36.976	1.6E-221
Rhizosphere	15	Resilience	Family	38.264	6.0E-227
Rhizosphere	16	Resilience	Family	8.208	3.7E-15
Rhizosphere	16	Resilience	OTU	9.592	2.3E-20
Rhizosphere	17	Resilience	OTU	8.124	7.3E-15
Rhizosphere	17	Resilience	Family	10.231	6.0E-23
Soil	3	Resistance	OTU	-1.567	7.4E-01
Soil	3	Resistance	Family	-1.655	6.3E-01
Soil	4	Resistance	Family	-3.040	2.2E-02
Soil	4	Resistance	OTU	-3.819	2.0E-03
Soil	5	Resistance	Family	0.573	1.0E+00
Soil	5	Resistance	OTU	0.818	1.0E+00
Soil	6	Resistance	OTU	-3.462	5.7E-03
Soil	6	Resistance	Family	-4.000	1.1E-03
Soil	7	Resistance	Family	-6.238	1.4E-06
Soil	7	Resistance	OTU	-7.440	3.5E-09
Soil	8	Resistance	Family	-4.605	1.4E-04
Soil	8	Resistance	OTU	-5.814	1.1E-06

Table S3 The number and proportion of network edge and vertices removed due to FDR correction

Network	Compartment	Treatment	Period	No.edges FDR	No.edges nFDR	Edges Removed	No.vertices FDR	No.vertices nFDR	Vertices Removed
Inter-Bac-Fung	Root	Stress	Drought	10	191	94.76%	17	177	90.40%
Cross-Bac-Fung	Root	Stress	Drought	95	1130	91.59%	102	540	81.11%
Bac-Bac	Root	Stress	Drought	77	888	91.33%	79	448	82.37%
Fung-Fung	Root	Stress	Drought	8	51	84.31%	13	49	73.47%
Bac-Bac	Soil	Stress	Drought	193	848	77.24%	187	611	69.39%
Inter-Bac-Fung	Soil	Stress	Drought	52	225	76.89%	73	257	71.60%
Cross-Bac-Fung	Soil	Stress	Drought	272	1164	76.63%	263	814	67.69%
Fung-Fung	Soil	Stress	Drought	27	91	70.33%	34	86	60.47%
Inter-Bac-Fung	Soil	Control	Drought	274	408	32.84%	226	309	26.86%
Inter-Bac-Fung	Rhizosphere	Stress	Drought	161	228	29.39%	143	185	22.70%
Cross-Bac-Fung	Rhizosphere	Stress	Drought	811	1085	25.25%	439	536	18.10%
Bac-Bac	Rhizosphere	Stress	Drought	481	643	25.19%	324	395	17.97%
Cross-Bac-Fung	Soil	Control	Drought	1859	2482	25.10%	788	972	18.93%
Bac-Bac	Soil	Control	Drought	1490	1956	23.82%	636	784	18.88%
Fung-Fung	Rhizosphere	Stress	Drought	169	214	21.03%	83	95	12.63%
Fung-Fung	Soil	Control	Drought	95	118	19.49%	74	83	10.84%
Bac-Bac	Leaf	Control	Drought	43	43	0	47	47	0
Bac-Bac	Leaf	Control	Rewetting	433	433	0	79	79	0
Bac-Bac	Leaf	Stress	Drought	141	141	0	93	93	0
Bac-Bac	Leaf	Stress	Rewetting	1015	1015	0	138	138	0
Bac-Bac	Rhizosphere	Control	Drought	10234	10234	0	887	887	0
Bac-Bac	Rhizosphere	Control	Rewetting	5050	5050	0	686	686	0

Bac-Bac	Rhizosphere	Stress	Rewetting	13730	13730	0	761	761	0
Bac-Bac	Root	Control	Drought	10518	10518	0	608	608	0
Bac-Bac	Root	Control	Rewetting	2755	2755	0	348	348	0
Bac-Bac	Root	Stress	Rewetting	9030	9030	0	495	495	0
Bac-Bac	Soil	Control	Rewetting	1151	1151	0	590	590	0
Bac-Bac	Soil	Stress	Rewetting	1879	1879	0	632	632	0
Cross-Bac-Fung	Leaf	Control	Drought	122	122	0	73	73	0
Cross-Bac-Fung	Leaf	Control	Rewetting	554	554	0	117	117	0
Cross-Bac-Fung	Leaf	Stress	Drought	189	189	0	117	117	0
Cross-Bac-Fung	Leaf	Stress	Rewetting	1436	1436	0	186	186	0
Cross-Bac-Fung	Rhizosphere	Control	Drought	11116	11116	0	1036	1036	0
Cross-Bac-Fung	Rhizosphere	Control	Rewetting	7371	7371	0	896	896	0
Cross-Bac-Fung	Rhizosphere	Stress	Rewetting	16408	16408	0	894	894	0
Cross-Bac-Fung	Root	Control	Drought	12684	12684	0	714	714	0
Cross-Bac-Fung	Root	Control	Rewetting	3478	3478	0	433	433	0
Cross-Bac-Fung	Root	Stress	Rewetting	11000	11000	0	596	596	0
Cross-Bac-Fung	Soil	Control	Rewetting	1505	1505	0	760	760	0
Cross-Bac-Fung	Soil	Stress	Rewetting	2127	2127	0	749	749	0
Inter-Bac-Fung	Leaf	Control	Drought	3	3	0	5	5	0
Inter-Bac-Fung	Leaf	Control	Rewetting	82	82	0	46	46	0
Inter-Bac-Fung	Leaf	Stress	Drought	2	2	0	4	4	0
Inter-Bac-Fung	Leaf	Stress	Rewetting	331	331	0	96	96	0
Inter-Bac-Fung	Rhizosphere	Control	Drought	777	777	0	391	391	0
Inter-Bac-Fung	Rhizosphere	Control	Rewetting	1529	1529	0	437	437	0
Inter-Bac-Fung	Rhizosphere	Stress	Rewetting	2398	2398	0	474	474	0
Inter-Bac-Fung	Root	Control	Drought	1840	1840	0	417	417	0
Inter-Bac-Fung	Root	Control	Rewetting	619	619	0	246	246	0
Inter-Bac-Fung	Root	Stress	Rewetting	1836	1836	0	409	409	0

Inter-Bac-Fung	Soil	Control	Rewetting	161	161	0	145	145	0
Inter-Bac-Fung	Soil	Stress	Rewetting	167	167	0	157	157	0
Fung-Fung	Leaf	Control	Drought	76	76	0	24	24	0
Fung-Fung	Leaf	Control	Rewetting	39	39	0	31	31	0
Fung-Fung	Leaf	Stress	Drought	46	46	0	22	22	0
Fung-Fung	Leaf	Stress	Rewetting	90	90	0	42	42	0
Fung-Fung	Rhizosphere	Control	Drought	105	105	0	77	77	0
Fung-Fung	Rhizosphere	Control	Rewetting	792	792	0	159	159	0
Fung-Fung	Rhizosphere	Stress	Rewetting	280	280	0	94	94	0
Fung-Fung	Root	Control	Drought	326	326	0	91	91	0
Fung-Fung	Root	Control	Rewetting	104	104	0	64	64	0
Fung-Fung	Root	Stress	Rewetting	134	134	0	69	69	0
Fung-Fung	Soil	Control	Rewetting	193	193	0	131	131	0
Fung-Fung	Soil	Stress	Rewetting	81	81	0	75	75	0

Table S4 Non-random topological features indicated by comparing empirical network against random networks (P values were calculated by one-sample t-test (two-sided) and adjusted by Bonferroni method)

Compartment	Treatment	Period	Network Indexes	Observation	Random network (mean ± sd)	P value
Root	Control	Drought	Average clustering coefficient	0.404	0.221 ± 0.012	6.22E-123
Root	Control	Rewetting	Average clustering coefficient	0.427	0.25 ± 0.013	4.54E-118
Root	Stress	Drought	Average clustering coefficient	0.094	0.016 ± 0.005	6.59E-124
Root	Stress	Rewetting	Average clustering coefficient	0.461	0.278 ± 0.012	6.22E-123
Root	Control	Drought	Average path distance	2.916	2.666 ± 0.024	1.35E-106
Root	Control	Rewetting	Average path distance	3.077	2.656 ± 0.027	6.89E-124
Root	Stress	Drought	Average path distance	6.065	4.533 ± 0.08	1.03E-132
Root	Stress	Rewetting	Average path distance	3.111	2.554 ± 0.021	1.04E-146
Root	Control	Drought	Transitivity	0.424	0.253 ± 0.006	8.48E-150
Root	Control	Rewetting	Transitivity	0.387	0.256 ± 0.007	1.01E-131
Root	Stress	Drought	Transitivity	0.29	0.03 ± 0.006	8.15E-168
Root	Stress	Rewetting	Transitivity	0.445	0.296 ± 0.006	7.06E-144
Rhizosphere	Control	Drought	Average clustering coefficient	0.267	0.074 ± 0.01	4.79E-133
Rhizosphere	Control	Rewetting	Average clustering coefficient	0.321	0.141 ± 0.011	5.84E-126
Rhizosphere	Stress	Drought	Average clustering coefficient	0.162	0.046 ± 0.007	1.68E-126
Rhizosphere	Stress	Rewetting	Average clustering coefficient	0.45	0.3 ± 0.013	5.63E-111
Rhizosphere	Control	Drought	Average path distance	4.449	3.261 ± 0.05	5.59E-142
Rhizosphere	Control	Rewetting	Average path distance	3.804	2.99 ± 0.034	2.63E-142
Rhizosphere	Stress	Drought	Average path distance	4.642	3.587 ± 0.042	2.27E-144
Rhizosphere	Stress	Rewetting	Average path distance	2.921	2.62 ± 0.022	2.81E-118
Rhizosphere	Control	Drought	Transitivity	0.283	0.098 ± 0.008	8.15E-141
Rhizosphere	Control	Rewetting	Transitivity	0.354	0.175 ± 0.008	2.13E-139
Rhizosphere	Stress	Drought	Transitivity	0.373	0.089 ± 0.007	5.54E-165

Rhizosphere	Stress	Rewetting	Transitivity	0.365	0.266 ± 0.006	2.58E-126
Soil	Control	Drought	Average clustering coefficient	0.175	0.041 ± 0.009	6.64E-122
Soil	Stress	Drought	Average clustering coefficient	0.135	0.011 ± 0.004	2.05E-153
Soil	Stress	Rewetting	Average clustering coefficient	0.166	0.017 ± 0.004	2.61E-161
Soil	Control	Rewetting	Average clustering coefficient	0.169	0.014 ± 0.004	5.25E-163
Soil	Control	Drought	Average path distance	4.586	3.664 ± 0.057	1.84E-125
Soil	Stress	Drought	Average path distance	6.039	4.72 ± 0.099	3.87E-117
Soil	Stress	Rewetting	Average path distance	5.734	4.254 ± 0.054	4.06E-148
Soil	Control	Rewetting	Average path distance	6.379	4.48 ± 0.049	5.17E-163
Soil	Control	Drought	Transitivity	0.374	0.067 ± 0.008	1.37E-162
Soil	Stress	Drought	Transitivity	0.249	0.022 ± 0.006	5.59E-162
Soil	Stress	Rewetting	Transitivity	0.266	0.028 ± 0.005	7.48E-172
Soil	Control	Rewetting	Transitivity	0.268	0.023 ± 0.004	1.08E-182
Leaf	Control	Rewetting	Average clustering coefficient	0.4	0.383 ± 0.018	4.96E-16
Leaf	Stress	Rewetting	Average clustering coefficient	0.377	0.338 ± 0.018	7.10E-42
Leaf	Control	Rewetting	Average path distance	4.093	2.492 ± 0.048	1.47E-156
Leaf	Stress	Rewetting	Average path distance	3.087	2.584 ± 0.034	1.25E-121
Leaf	Control	Rewetting	Transitivity	0.644	0.483 ± 0.012	1.94E-117
Leaf	Stress	Rewetting	Transitivity	0.536	0.399 ± 0.01	2.47E-118

Random networks were generated at the Molecular Ecological Network Analyses Pipeline (MENAP) by randomly rewiring all the links while keeping the numbers of nodes and links of the empirical network.

References

1 Coenen, A. R., Hu, S. K., Luo, E., Muratore, D. & Weitz, J. S. A primer for microbiome time-series analysis. *Front. Genet.* **11** (2020).

2 de Vries, F. T. *et al.* Soil bacterial networks are less stable under drought than fungal networks. *Nat. Commun.* **9**, 3033 (2018).

3 Barnard, R. L., Osborne, C. A. & Firestone, M. K. Responses of soil bacterial and fungal communities to extreme desiccation and rewetting. *ISME J.* **7**, 2229-2241 (2013).

4 Barnard, R. L., Osborne, C. A. & Firestone, M. K. Changing precipitation pattern alters soil microbial community response to wet-up under a Mediterranean-type climate. *The ISME Journal* **9**, 946-957 (2015).

5 Ochoa-Hueso, R. *et al.* Drought consistently alters the composition of soil fungal and bacterial communities in grasslands from two continents. *Global Change Biology* **24**, 2818-2827 (2018).

6 McHugh, T. A., Koch, G. W. & Schwartz, E. Minor changes in soil bacterial and fungal community composition occur in response to monsoon precipitation in a semiarid grassland. *Microb. Ecol.* **68**, 370-378 (2014).

7 de Vries, F. T. & Shade, A. Controls on soil microbial community stability under climate change. *Front Microbiol* **4**, 265 (2013).

8 de Vries, F. T. *et al.* Land use alters the resistance and resilience of soil food webs to drought. *Nat. Clim. Chang.* **2**, 276 (2012).

9 de Vries, F. T. *et al.* Legacy effects of drought on plant growth and the soil food web. *Oecologia* **170**, 821-833 (2012).
