# Supplementary material for: Co-occurrence networks reveal more complexity than community composition in resistance and resilience of microbial communities
Source: Nat Commun. 2022 Jul 5;13:3867. doi: 10.1038/s41467-022-31343-y (PMC9256619; doi:10.1038/s41467-022-31343-y)
Supplement: Supplementary file 2 — Reporting Summary [file 41467_2022_31343_MOESM2_ESM.pdf]

nature research

Corresponding author(s): John Taylor

Last updated by author(s): 2022/6/2

Reporting Summary

Nature Research wishes to improve the reproducibility of the work that we publish. This form provides structure for consistency and transparency in reporting. For further information on Nature Research policies, see our Editorial Policies and the Editorial Policy Checklist.

Statistics

For all statistical analyses, confirm that the following items are present in the figure legend, table legend, main text, or Methods section.

n/a	Confirmed	
☐	☒	The exact sample size (*n*) for each experimental group/condition, given as a discrete number and unit of measurement
☐	☒	A statement on whether measurements were taken from distinct samples or whether the same sample was measured repeatedly
☐	☒	The statistical test(s) used AND whether they are one- or two-sided<br>*Only common tests should be described solely by name; describe more complex techniques in the Methods section.*
☒	☐	A description of all covariates tested
☐	☒	A description of any assumptions or corrections, such as tests of normality and adjustment for multiple comparisons
☐	☒	A full description of the statistical parameters including central tendency (e.g. means) or other basic estimates (e.g. regression coefficient) AND variation (e.g. standard deviation) or associated estimates of uncertainty (e.g. confidence intervals)
☐	☒	For null hypothesis testing, the test statistic (e.g. *F*, *t*, *r*) with confidence intervals, effect sizes, degrees of freedom and *P* value noted<br>*Give P values as exact values whenever suitable.*
☒	☐	For Bayesian analysis, information on the choice of priors and Markov chain Monte Carlo settings
☒	☐	For hierarchical and complex designs, identification of the appropriate level for tests and full reporting of outcomes
☐	☒	Estimates of effect sizes (e.g. Cohen's *d*, Pearson's *r*), indicating how they were calculated

Our web collection on statistics for biologists contains articles on many of the points above.

Software and code

Policy information about availability of computer code

Data collection	No software was used for data collection
Data analysis	vegan 2.6.2, igraph 1.3.1, lme4 version 1.1.29, ggplot2 version 3.3.6, psych 2.2.5, R version 4.2.0

For manuscripts utilizing custom algorithms or software that are central to the research but not yet described in published literature, software must be made available to editors and reviewers. We strongly encourage code deposition in a community repository (e.g. GitHub). See the Nature Research guidelines for submitting code & software for further information.

Data

Policy information about availability of data

All manuscripts must include a data availability statement. This statement should provide the following information, where applicable:

- Accession codes, unique identifiers, or web links for publicly available datasets
- A list of figures that have associated raw data
- A description of any restrictions on data availability

No new data were generated in this study. The data used in this study had been deposited in the Sequence Read Archive database under accession code PRJNA412410 [https://www.ncbi.nlm.nih.gov/bioproject/PRJNA412410], PRJNA494573 [https://www.ncbi.nlm.nih.gov/bioproject/PRJNA494573], PRJNA435634 [https://www.ncbi.nlm.nih.gov/bioproject/PRJNA435634], PRJNA435642 [https://www.ncbi.nlm.nih.gov/bioproject/PRJNA435642], and PRJNA435643 [https://www.ncbi.nlm.nih.gov/bioproject/PRJNA435643]. Source data are provided as a Source Data file.

Field-specific reporting

Please select the one below that is the best fit for your research. If you are not sure, read the appropriate sections before making your selection.

☐ Life sciences ☐ Behavioural & social sciences ☒ Ecological, evolutionary & environmental sciences

For a reference copy of the document with all sections, see nature.com/documents/nr-reporting-summary-flat.pdf

Ecological, evolutionary & environmental sciences study design

All studies must disclose on these points even when the disclosure is negative.

Study description	Our research had three blocks each consisted of 6 randomly distributed plots representing an full factorial interaction of three treatments and two sorghum cultivars. We collected root, rhizosphere, soil and leaf samples weekly over 17 weeks. For each sample, we used amplicon sequencing to detect bacterial and fungal communities
Research sample	We collected 1026 sample from three treatments, two sorghum cultivars, 17 time points and four compartments.<br>Three treatments: control, pre-flowering drought and post-flowering drought; Two sorghum cultivars: the pre-flowering, drought tolerant sorghum cultivar RTx430, and the post-flowering, drought tolerant (or 'stay green') cultivar BTx642; Four compartments: Leaf, Root, Rhizosphere, and Soil
Sampling strategy	For each plot at each sampling date, at least ten individual sorghum plants were removed to collect leaf, root and rhizosphere samples. The 3rd and 4th youngest, fully expanded leaves of the ten plants were collected as a leaf sample. Roots removed from the ten plants were washed with phosphate buffer, and the liquids were centrifuged to collect a rhizosphere sample. Soil was collected adjacent to the ten sampled plants using soil collection tubes. The rationale for sampling at least ten sorghum individuals is to satisfy the analysis of multi-Omics, and this sample size has been proven to be sufficient for the analysis of amplicon and metagenomic sequencing, transcriptome, and metabolome, etc.
Data collection	DNA was extracted from 1026 samples. ITS2 and 16S was amplified and subject to library preparation. Libraries were sequenced by Illumina Miseq PE300 at the Vincent J. Coates Genomics Sequencing Laboratory (GSL, University of California, Berkeley, CA, USA). Raw sequencing were filtered and clustered into OTUs using Usearch. Ling Xu, Cheng Gao, Liliam Montoya extracted DNAs, library preparation and sequencing. Cheng Gao and Ling Xu carried out the Bioinformatic analysis.
Timing and spatial scale	Weekly samples of leaf, root, rhizosphere and soil were taken in 2016 for control plots on June 8, 15, 22, 29; July 6, 13, 20, 27; August 3, 10, 17, 24, 31, and September 7, 14, 21, 28. Our time scale covered the life cycle of sorghum from seedling to maturation.<br>Our research has 18 plots (16m * 8m each) in a 76 m * 56 m field.
Data exclusions	No data was excluded from the analysis
Reproducibility	Our experiment used three replicate block of each treatment, and we confirm that all attempts at replications were successful.
Randomization	Our experiment is a random block design of three replicates.
Blinding	Blinding is not possible for our research as obvious drought-related plant phenotypes are visible to collectors at sampling stage

Did the study involve field work? ☒ Yes ☐ No

Field work, collection and transport

Field conditions	Our agricultural filed is located in the Central Valley, a semiarid zone with a mean annual temperature of 17.8°C and mean annual precipitation of 325 mm, almost all of which falls between November and April.
Location	36.6008° N, 119.5109° W
Access & import/export	No permit is required to work on sorghum in California
Disturbance	No disturbance to natural environment

Reporting for specific materials, systems and methods

We require information from authors about some types of materials, experimental systems and methods used in many studies. Here, indicate whether each material, system or method listed is relevant to your study. If you are not sure if a list item applies to your research, read the appropriate section before selecting a response.

Materials & experimental systems

n/a	Involved in the study	
☒	☐	Antibodies
☒	☐	Eukaryotic cell lines
☒	☐	Palaeontology and archaeology
☒	☐	Animals and other organisms
☒	☐	Human research participants
☒	☐	Clinical data
☒	☐	Dual use research of concern

Methods

n/a	Involved in the study	
☒	☐	ChIP-seq
☒	☐	Flow cytometry
☒	☐	MRI-based neuroimaging
