# Supplementary material for: Co-occurrence networks reveal more complexity than community composition in resistance and resilience of microbial communities
Source: Nat Commun. 2022 Jul 5;13:3867. doi: 10.1038/s41467-022-31343-y (PMC9256619; doi:10.1038/s41467-022-31343-y)
Supplement: Supplementary file 4 — Description of Additional Supplementary Files [file 41467_2022_31343_MOESM4_ESM.docx]

File Name: Source Data

Description: Source data for the Figures 1-5.

File Name: Supplementary Data 1

Description: List of network hubs detected in this study
